# RNA-sequencing Identifies Novel Pathways in Sarcoidosis Monocytes

**DOI:** 10.1038/s41598-017-02941-4

**Published:** 2017-06-02

**Authors:** Jaya Talreja, Pershang Farshi, Adnan Alazizi, Francesca Luca, Roger Pique-Regi, Lobelia Samavati

**Affiliations:** 1 Department of Internal Medicine, Division of Pulmonary, Critical Care and Sleep Medicine, Wayne State University School of Medicine and Detroit Medical Center, Detroit, MI 48201 USA; 2 Center for Molecular Medicine and Genetics, Wayne State University School of Medicine, 540 E. Canfield, Detroit, MI 48201 USA; 3 Department of Obstetrics and Gynecology, Wayne State University School of Medicine, Detroit, MI 48201 USA

## Abstract

Sarcoidosis is a complex systemic granulomatous disorder of unknown etiology. Genome-wide association studies have not been able to explain a causative role for nucleotide variation in its pathogenesis. The goal of the present study was to identify the gene expression profile and the cellular pathways altered in sarcoidosis monocytes via RNA-sequencing. Peripheral blood monocytes play a role in sarcoidosis inflammation. Therefore, we determined and compared the transcriptional signature of monocytes from peripheral blood from sarcoidosis patients and healthy controls via RNA-sequencing. We found 2,446 differentially expressed (DE) genes between sarcoidosis and healthy control monocytes. Analysis of these DE genes showed enrichment for ribosome, phagocytosis, lysosome, proteasome, oxidative phosphorylation and metabolic pathways. RNA-sequencing identified upregulation of genes involved in phagocytosis and lysosomal pathway in sarcoidosis monocytes, whereas genes involved in proteasome degradation and ribosomal pathways were downregulated. Further studies are needed to investigate the role of specific genes involved in the identified pathways and their possible interaction leading to sarcoidosis pathology.

## Introduction

Sarcoidosis is a multisystem granulomatous disease of unknown etiology characterized by granuloma formation in barrier organs such as lungs, skin, and eyes. It results in significant morbidity and mortality, primarily from respiratory failure in the US^1–3^. It is thought that exposure to the airborne antigens in a genetically susceptible host leads to disease initiation^2–5^. Granulomatous inflammation in sarcoidosis is characterized by activated macrophages as well as CD4^+^ T cell infiltration with predominant Th1 cytokine expression^6, 7^.

Several studies attempted to identify genetic risk of developing sarcoidosis using various techniques such as a candidate gene approach, microarray of candidate genes, and genetic linkage analysis^3, 8, 9^. Several genes were linked to sarcoidosis susceptibility including major histocompatibility complex (MHC) and HLA antigens class I ﻿and II such as loci HLA-B8 and HLA-DRB1^3, 8, 10^. Similar candidate gene approaches additionally identified susceptibility genes involved in antigen processing, antigen presentation, macrophage and T-cell activation, and genes involved in injury repair^11–14^.

The similarities of sarcoidosis to infectious granulomatous diseases and its association with major histocompatibility loci suggest a major role for inciting microbial triggers^15, 16^. The presence of activated macrophages and the expansion of oligoclonal T cells and B cells suggest an important role of immunity in this disease^2, 17^. Several lines of evidence indicate that aberrant functioning of macrophages, dendritic cells, and monocytes may underlie the Th1 skewness in sarcoidosis^18, 19^. Similarly, previous studies from our laboratory demonstrated that sustained activation of p38 MAPK is associated with a lack of a negative feedback loop through kinase phosphatase (MKP)-1 leading to persistent inflammation in macrophages^19, 20^. Inhibition of inflammatory signaling in macrophages and monocytes led to modulation of activated T cells and their responses to mitogens^19^.

Our goal was to unravel the transcriptional signature, and to identify the genes and associated pathways that may be dysregulated in monocytes in sarcoidosis. To this end we interrogated the poly-adenylated fraction of the transcriptome via RNA-sequencing, thus reaching unprecedented depth in the analysis of the gene regulation events that are altered. To our knowledge, this is the first RNA-seq study to compare the transcriptional signature of peripheral blood monocytes from sarcoidosis patients and healthy controls.

## Results

### Differential gene expression and pathway analysis in monocytes

The study included two groups comprising of 20 patients with sarcoidosis and 20 healthy controls. The subject demographics are displayed in Table 1. There was no significant difference in age and BMI between patients and healthy controls (p > 0.05). To reduce the effect of potential confounding factors associated with variation in ancestry proportions, we included subjects with the same self-reported race. All subjects were non-smokers, and none was on immune-suppressive medication. All sarcoidosis subjects had lung involvement with chest radiograph stage 2 or 3. We prepared RNA-seq libraries from mRNA isolated from the peripheral blood monocytes obtained from sarcoidosis patients and healthy controls. The schematic study design of RNA-seq library preparation, work flow and analysis are shown in Supplementary Fig. S1. Alignment of the filtered reads to the reference human genome hg19 showed about 90% total aligned reads per RNA-seq library sample.

**Table 1:** Subject Demographics

Characteristic	Patients	Control Subjects
**Age** (year)	46.11 ± 14.4	48.7 ± 13.4
**BMI**	29 ± 10.4	28 ± 3.6
**Gender**, **N** (**%**)		
Female	9 (45)	10 (50)
Male	11 (55)	10 (50)
**Self-reported Race**, **N** (**%**)		
African American	20(100)	20 (100)
White	0 (0)	0 (0)
**CXR stage**, **N** (**%**)		
0	0 (0)	NA
1	0 (0)	NA
2	6 (60)	NA
3	4 (40)	NA
**Organ Involvements**, **N** (**%**)		
Neuro-ophtalmologic	2(10)	NA
Lung	20 (100)	NA
Skin	2 (10)	NA
Multiorgans	15 (75)	NA
**PPD**	Negative	Negative
**AFB/culture**	Negative	NA
**IGRA**	Negative	NA

Definition of abbreviations: BMI = body mass index, CXR = chest X-ray, NA = not applicable, PPD = purified protein derivative, AFB = acid-fast bacilli, IGRA = interferon gamma release assay.

**Figure 1:**
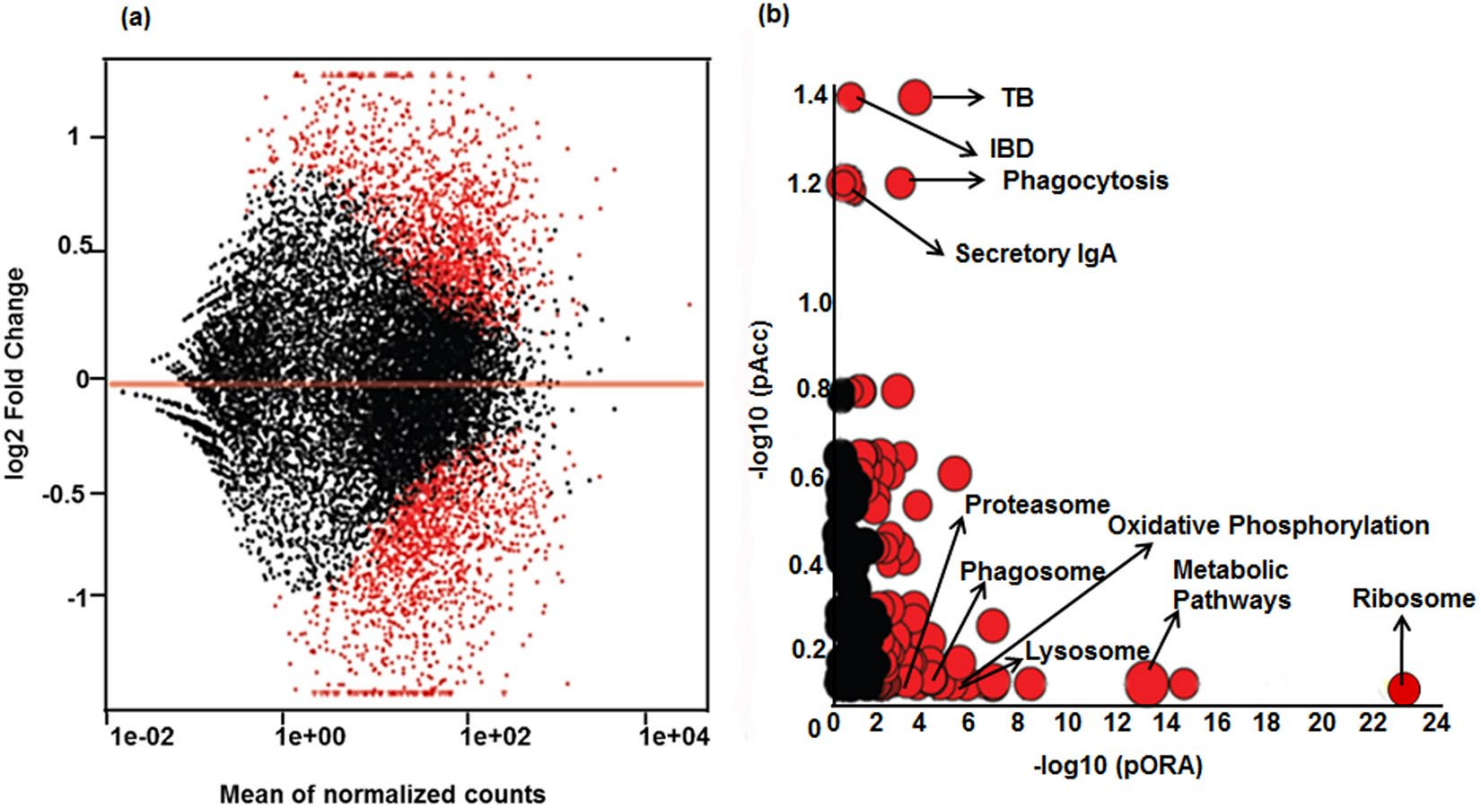
Differential gene expression and pathways between control and sarcoidosis monocytes. (**a**) Scatter plot of the entire gene expression data (healthy versus sarcoidosis monocytes) analyzed by DEseq2 analysis tool, where the log_2_-fold change (>0.5) of each gene is plotted against the total number of counts recorded for that gene. DE genes (FDR < 5%) are highlighted in red. (**b**) Significant pathways modulated in sarcoidosis monocytes as compared to healthy controls. Pathway analysis was done on the DE genes (log_2_-fold change > 0.6 and FDR < 5%) using iPathwayGuide analysis tool that uses two types of evidence: the over-representation on the horizontal axis (pORA) and the perturbation on the vertical axis (pAcc). Significant pathways (FDR < 5%) are shown in red, whereas non-significant are in black. The size of the circle is proportional to the number of genes in that pathway.

First, we compared the DE genes between the healthy control and sarcoidosis monocytes using DEseq2. Figure 1a shows the distribution of log_2_-fold change and expression level for the 8,069 genes expressed in these samples. The DE genes (2,446; FDR < 5%) between sarcoidosis and healthy control monocytes are highlighted in red. Among DE genes, 1,221 were downregulated whereas 1,225 were upregulated in sarcoidosis monocytes. To determine the pathways that are modulated in the sarcoidosis monocytes compared to healthy control monocytes, we performed KEGG pathway analysis using iPathwayGuide and the Gene trail tool on the DE genes (log_2_-fold change of 0.6 and FDR < 5%). Figure 1b shows the score plot of pathways using impact analysis. Impact analysis uses two types of evidence: i) the over-representation of DE genes in a given pathway and ii) the perturbation of that pathway computed by propagating the measured expression changes across the pathway topology^21–23^. The pathway topologies consist of genes and their interactions that are obtained from the KEGG database^24–27^. Significant pathways with FDR < 5% are shown in red and non-significant in black (Fig.1b). The size of the circle is proportional to the number of genes involved in that pathway. Topological analysis identified 103 pathways significantly enriched in sarcoidosis as compared to healthy controls. The most significant pathways were: ribosome (FDR = 1 × 10^−24^), metabolic pathways (FDR = 5.1 × 10^−14^), oxidative phosphorylation (FDR = 4.0 × 10^−6^), lysosome (FDR = 1.7 × 10^−5^), FcγR-mediated phagocytosis (FDR = 1.5 × 10^−4^), phagosome (FDR = 1.9 × 10^−2^), and proteasome (FDR = 1.9 × 10^−2^).

**Figure 2:**
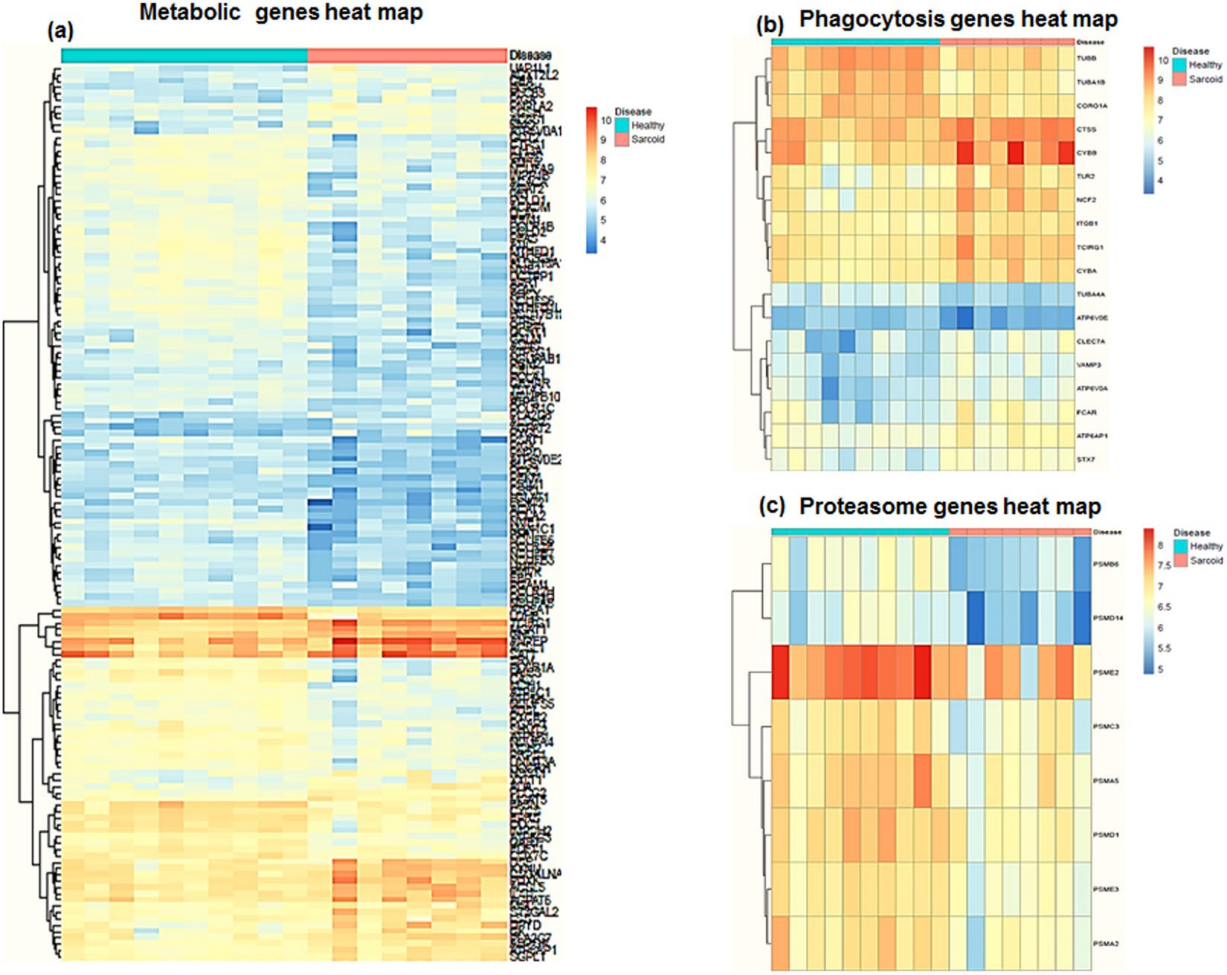
Heat maps of DE genes of three significant pathways in sarcoidosis versus healthy monocytes. (**a**) Heatmap of the 143 genes involved in metabolic pathways between two groups. (**b**) Heatmaps of 19 genes involved in phagocytosis and (**c**) Heatmaps of 9 genes involved in proteasome (log_2_-fold change > 0.5 & FDR < 5%). Dendrograms according to means identifying genes levels in all three pathways show two distinct clusters. Red shade represents high expression and blue shade represents low expression.

Furthermore, pathway analysis identified that genes involved in monocyte activation, inflammation, and innate and adaptive immune responses were highly enriched in sarcoidosis monocytes. Especially, gene expression was increased in sarcoidosis monocytes for various pathways involved in phagosome, lysosome, monocyte activation, and leukocyte migration and trafficking. In contrast, genes involved in proteasome and ribosomal biogenesis were found to be downregulated in sarcoidosis monocytes as compared to control monocytes. Figure 2 shows the heat-map of sarcoidosis DE genes involved in three of the significant (FDR < 5%) pathways: (a) metabolic pathway (most of the genes involved in this pathway were downregulated), (b) phagocytosis (most of the genes involved in this pathway were upregulated), (c) proteasome pathway (genes involved in this pathway were found to be downregulated).

**Figure 3:**
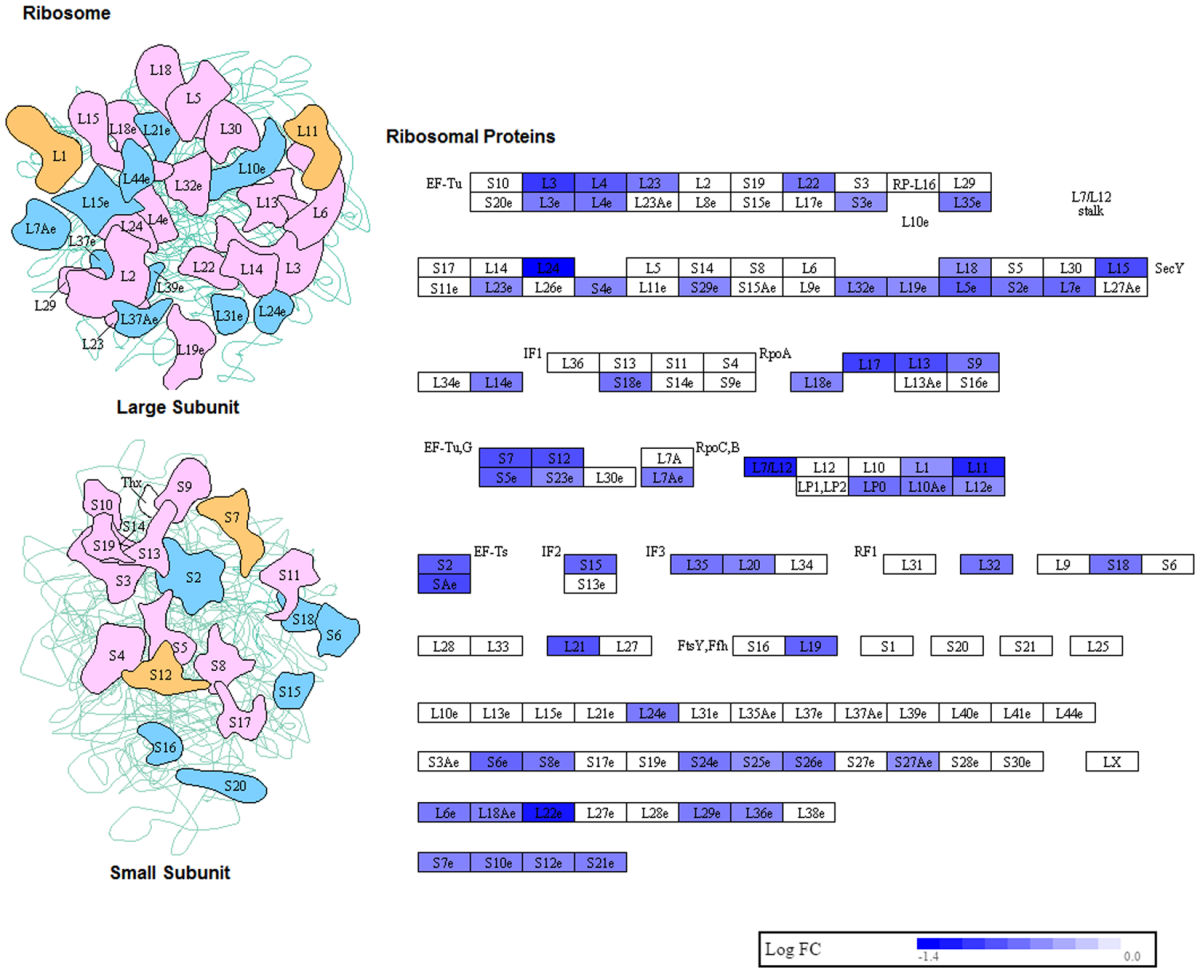
Downregulation of genes involved in ribosomal pathway in sarcoidosis monocytes. Graphic illustration of pathway analysis of DE (log_2_-fold change with FDR < 0.05) genes related to ribosome in sarcoidosis monocytes. The pathway diagram is overlaid with the computed perturbation of each gene. The perturbation accounts both for the gene’s measured fold changes and for accumulated perturbation propagated from any upstream genes (accumulation). The color intensity corresponds to the level of upregulation (red) or downregulation (blue) of the DE genes in sarcoidosis monocytes versus healthy monocytes. Note: A number of genes encoding large and small ribosomal subunits were downregulated.

**Figure 4:**
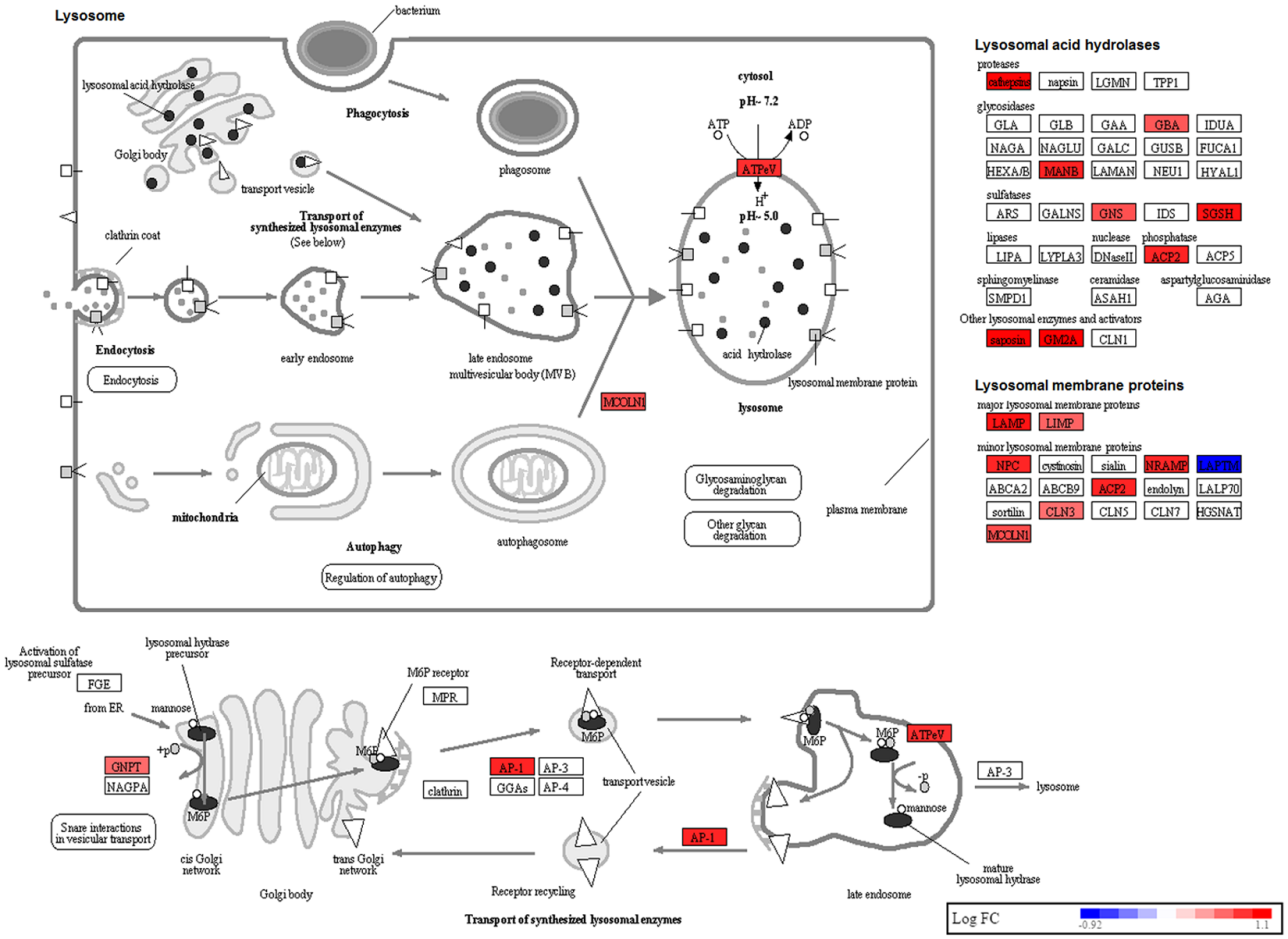
Upregulation of genes involved in lysosomal pathway in sarcoidosis monocytes. Graphic illustration of pathway analysis of DE (log_2_-fold change with FDR < 0.05) genes related to lysosome in sarcoidosis monocytes. The pathway diagram is overlaid with the computed perturbation of each gene. The perturbation accounts both for the gene’s measured fold changes and for accumulated perturbation propagated from any upstream genes (accumulation). The color intensity corresponds to the level of upregulation (red) or downregulation (blue) of the DE genes in sarcoidosis monocytes versus healthy monocytes. Note: Various genes involved in lysosomal membrane proteins and acid hydrolases were upregulated except LAPTM4B gene which was downregulated.

**Figure 5:**
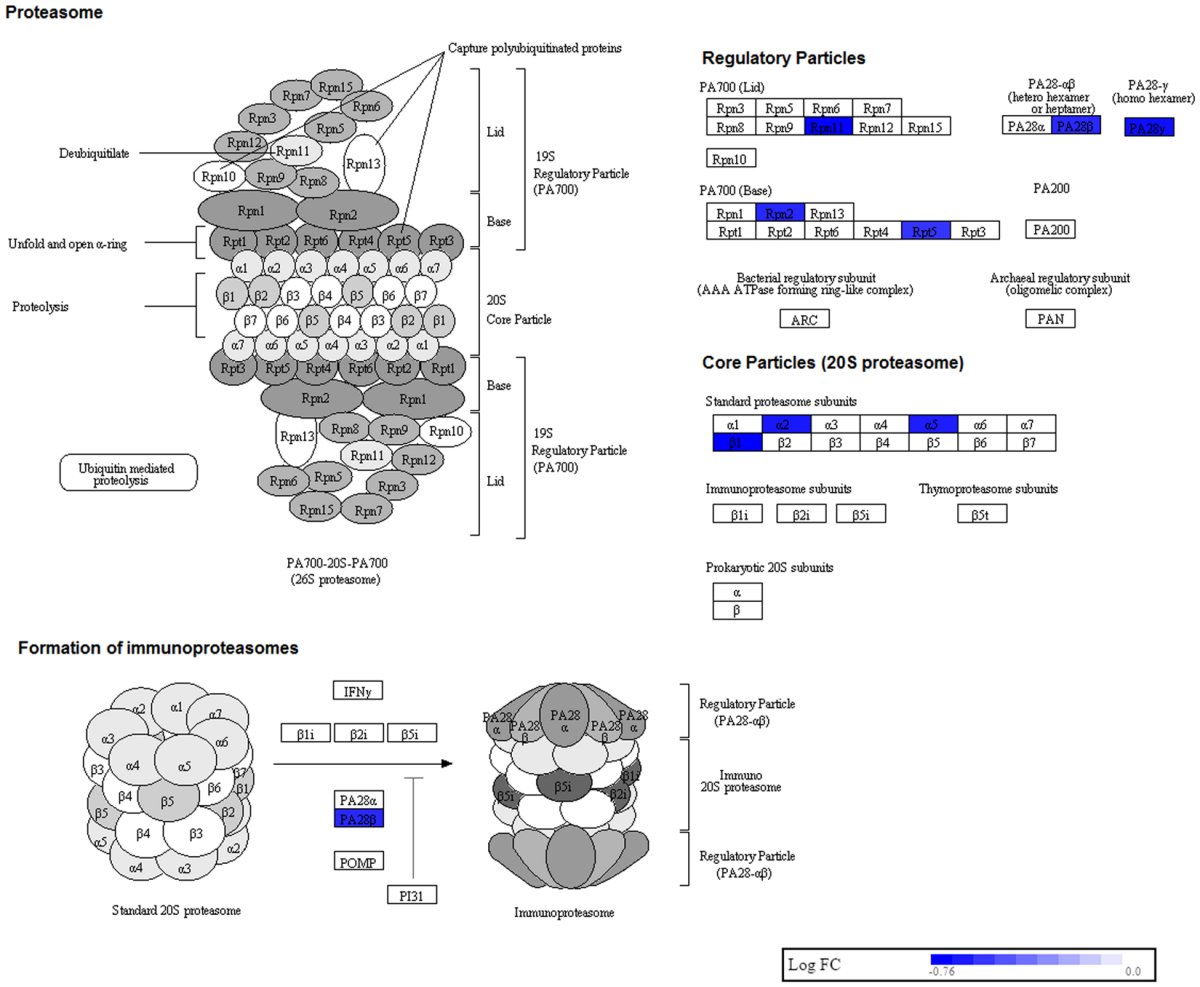
Proteasome in sarcoidosis monocytes. Graphic illustration of pathway analysis of DE (log_2_-fold change with FDR < 0.05) genes related to proteasome and immunoproteasome in sarcoidosis monocytes. The pathway diagram is overlaid with the computed perturbation of each gene. The perturbation accounts both for the gene’s measured fold changes and for accumulated perturbation propagated from any upstream genes (accumulation). Note: Several genes involved in regulatory and core particles and immunoproteasomes were downregulated (blue) in sarcoidosis monocytes.

The top enriched pathway was ribosome pathway (Fig. 3). The genes involved in this pathway were downregulated as compared to control monocytes. Figure 4 shows lysosome pathway that was highly enriched in sarcoidosis monocytes. Most of genes related to lysosomal acid hydrolases and lysosomal membrane proteins were found to be upregulated. Whereas, the genes involved in proteasome pathway were found to be downregulated (Fig. 5). The genes involved in the assembly of regulatory and core particle of proteasome were downregulated. The immunoproteasome gene PA28γ (also known as PSME3) that encodes the gamma subunit of 11 S regulator of immunoproteasome was downregulated in sarcoidosis monocytes.

### Validation of RNA-seq data via qRT-PCR

To validate the RNA-seq data, we evaluated the expression levels of selected DE genes. RNA was isolated from the monocytes obtained from an independent set of sarcoidosis patients (n = 10) and healthy controls (n = 10) and was subjected to qRT-PCR using selected primers. Our RNA-seq data showed that the phagocytosis, oxidative phosphorylation, ribosomal, and inflammatory pathways are impacted in sarcoidosis. Among DE genes involved in phagocytosis, we chose the gene encoding ATPase H^+^ transporting accessory protein 1 (ATP6AP1) for validation. This gene encodes an essential multitasking protein important for housekeeping acidification of membrane-bound compartments including acidifying endosomes, lysosomes, phagosomes, compartments for uncoupling receptors, and autophagosomes and it is involved in oxidative phosphorylation^28^. We selected the gene encoding the lysosomal membrane proteins (LAMP)-2, which is integral part of lysosomal membrane^29^ and was highly expressed in sarcoidosis. Furthermore, we chose the gene encoding cytochrome B-245 β chain that is an integral part of oxidative phosphorylation and its variation has been reported in chronic granulomatous disease^30^. Serpin family member 1 (SERPINA1) encodes peptidase inhibitor clade 1, which is α-antitrypsin precursor^31^. We also chose two genes from ribosomal pathways for validation. Genes encoding the protein SA of 40S subunit (RPSA), which is involved in ribosomal assembly, and the ribosomal protein L10a (RPL10A), which is the component of 60S subunit were selected for further validation.

**Figure 6:**
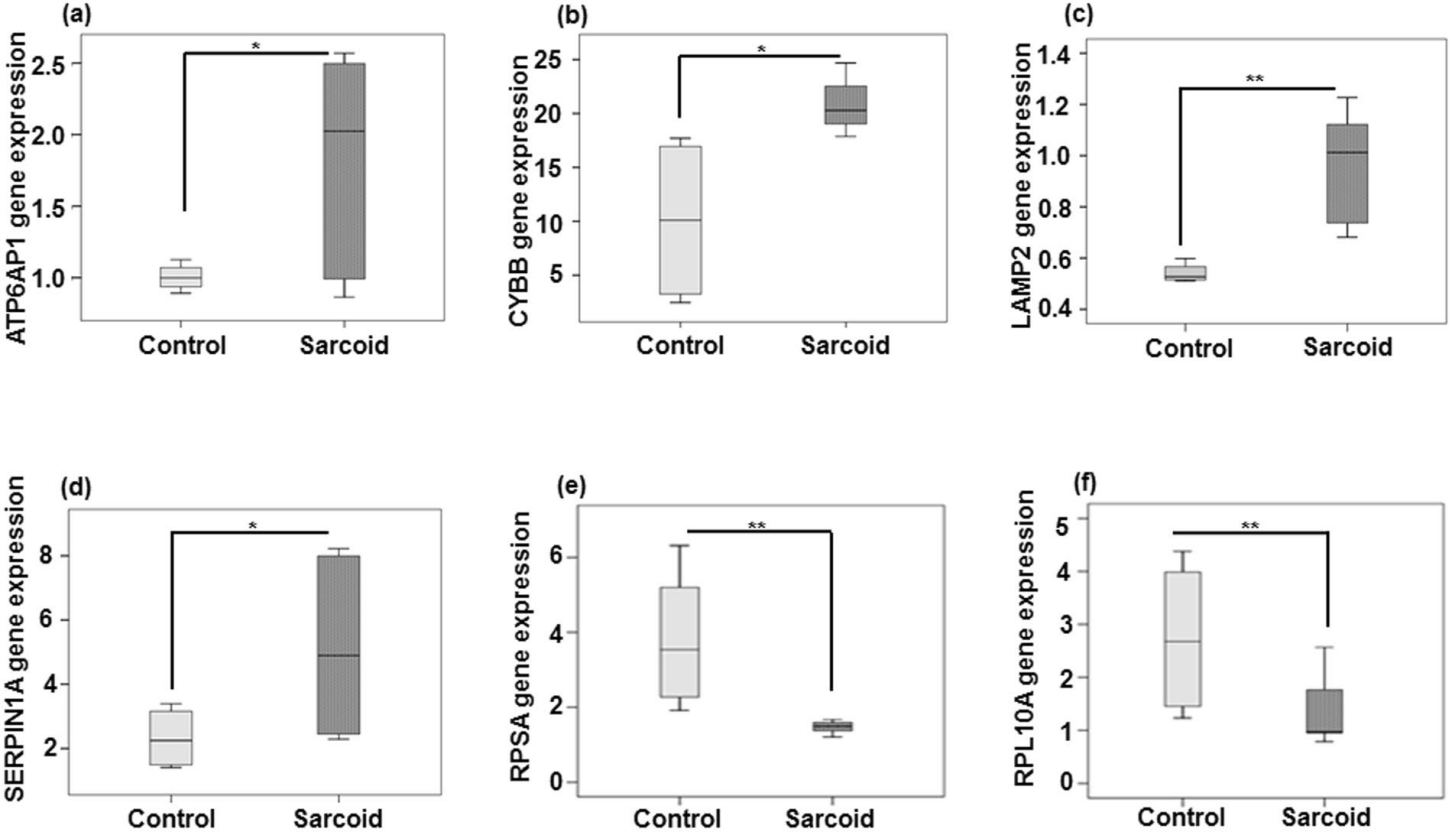
Validation of RNA-seq data by qRT-PCR. Total RNA was extracted from the monocytes from independent sets of 10 sarcoidosis patients and 10 healthy controls. Isolated RNAs were and reverse-transcribed using the Reverse Transcription System. The primers targeted (**a**) ATP6AP1, (**b**) CYBB, (**c**) LAMP2, (**d**) SERPIN1A, (**e**) RPSA and (**f**) RPL10A to amplify cDNA using iQSYBR Green Supermix. Relative mRNA levels were calculated by normalizing to β-actin. Box plots represent the normalized expression level of each gene of monocytes of healthy controls and sarcoidosis monocytes. Data were analyzed using the paired, two-tailed Student’s *t* test and the results were expressed as fold change. *Represents a p value < 0.05 and **signifies a p < 0.001.

We validated selected genes from those pathways using qRT-PCR using primers for the following genes, ATP6AP1, LAMP2, CYBB, SERPINA1, RPSA and RPL10A. The relative gene expression levels for ATP6AP1, CYBB, LAMP2, and SERPINA1 were significantly higher in sarcoidosis monocytes as compared to healthy monocytes (Fig. 6a–d). Additionally, both RPSA and RPL10A genes encoding ribosomal proteins were found to be significantly downregulated in sarcoidosis monocytes as compared to healthy monocytes (Fig. 6e and f). The relative gene expression data are presented as box plots as mean ± SEM from ten subjects with sarcoidosis monocytes and ten healthy controls monocytes. *Represents a p value < 0.05 and **a p < 0.001 using a paired student *t*-test. These qRT-PCR data confirm in independent sets of patients and controls the RNA- seq data.

## Discussion

It is well accepted that sarcoidosis is a polygenic and multifactorial disease^32^. Numerous association studies attempted to identify genetic susceptibility in sarcoidosis and various genes were found to increase the risk of developing this disease, among which the chemokine receptors, the tumor necrosis factor (TNF)-α, and several HLA loci and MHC class II antigens^3, 32^. Despite identifying some genes that may contribute to the risk of developing sarcoidosis or modify its severity^3, 33^, none have fully explained the complex nature of this disease. Several studies using heterogeneous samples, including peripheral blood, tissue samples or BALs, attempted to identify a gene signature in this disease^34–36^. Here, we investigated and compared the transcriptional profile of monocytes, one important player in the formation of multi-nucleated giant cells in sarcoidosis granuloma^37^. Our aim was to determine the transcriptional responses of peripheral blood monocytes and to unravel the cellular mechanisms responsible for the immunopathogenesis of sarcoidosis.

We found that 2,446 genes are significantly differentially expressed in monocytes between sarcoidosis and healthy controls. Functional analysis of DE genes showed the enrichment for various critical cellular pathways including ribosome and metabolic pathways, the phagosome and phagocytosis, lysosome, oxidative phosphorylation, proteasome, purine metabolism and cellular pathways involved in the inflammatory, innate and adaptive immune response.

One of the highly significant processes was the ribosomal pathway (FDR = 1 × 10^−24^). In sarcoidosis monocytes 62 genes related to ribosome were significantly downregulated compared to control monocytes (Fig. 3). In contrast, genes encoding mitochondrial ribosomes were upregulated. This might be due to different regulation between mitochondrial and cytosolic ribosomes. The biogenesis of ribosomes is an extremely energetically expensive cellular process that has long been linked to human health and disease. More recently, it has been shown that ribosome biogenesis is intimately linked to multiple cellular signaling pathways and that defects in ribosome production can lead to a wide variety of human diseases^38^. This novel pathway previously has not been described in sarcoidosis. Further studies are needed to determine the functional contribution of ribosomal pathways in sarcoidosis pathology.

We found about 50 DE genes that are involved in phagosome and phagocytosis in sarcoidosis monocytes compared to control monocytes. Among these genes, several were related to pattern recognition molecules, inflammatory signaling, leukocyte migration, and trafficking. In particular, genes encoding TLR2, RIPK2, CLEC7A, NLRP3 and MAP2K1 were upregulated. Similarly, we found genes involved in cytokine-cytokine receptor (CCR) signaling, and lipid metabolism that were aberrant in sarcoidosis^35, 39, 40^.

Another important pathway related to sarcoidosis pathology is the lysosomal pathway^5^. We identified a large number of genes involved in lysosomal acid hydrolases, lysosomal membrane proteins and various genes related to the function of lysosomes such as the V-ATPase complex that were upregulated in sarcoidosis monocytes as compared to healthy monocytes. Lysosomes are subcellular organelles found in all animal cells that digest cellular debris, damaged organelles and invading microorganisms. Lysosomal membrane proteins maintain the integrity of lysosomes and regulate lysosomal trafficking, fusion and intralysosomal pH. Aberrant lysosomal function in sarcoidosis has long been postulated^41^. One conventional immune modulatory therapies in sarcoidosis is Chloroquine^42^ which has been shown to inhibit lysosomal function and modulates autophagy^43^. Surprisingly, the LAPTM4B gene, which is involved in lysosomal machinery and has been shown to interact with ceramide to facilitate its removal from the late endosomal organelles was downregulated^44^ (Fig. 4). In contrast to genes involved in lysosomal pathways, genes involved in proteasome and immune-proteosome assembly were found to be downregulated in sarcoidosis monocytes (Fig. 5). Proteasomes are macromolecular proteins that are responsible for degradation of intracellular proteins in an ATP dependent manner^45^. Proteasomes degrade regulatory proteins and misfolded or damaged proteins^45^. Along with lysosomal function, proteasomes play a crucial role in protein homeostasis.

We identified several major dysregulated pathways related to metabolism and oxidative phosphorylation in sarcoidosis monocytes. There were 143 differentially expressed genes related to metabolism. Among genes involved in metabolism, we found a predominant downregulation of genes involved in purine metabolism (FDR = 2.9 × 10^−7^). A major set of metabolic genes was involved in oxidative phosphorylation and mitochondrial electron transfer chain. Recently, it has been recognized that metabolic reprogramming is a prerequisite for inflammation and immunological responses to diverse stimuli, both in macrophages and lymphocytes^46–48^. However, most of these studies were done in animal models or isolated macrophages and lymphocytes^46^. In the current study, we also found several differentially expressed genes involved in fatty acid and cholesterol metabolism in sarcoidosis monocytes.

We found that various genes involved in cholesterol synthesis and β-oxidation pathways, including acetyl CoA transferases, were lower expressed in sarcoidosis monocytes as compared to healthy monocytes. Other genes involved in lipid metabolisms, including several genes involved in mitochondrial β-oxidation (e.g. various isoforms of Acetyl-Coenzyme A acetyltransferases (ACATs) appear to be unique in sarcoidosis monocytes. No previous studies have identified these genes being associated with the differentiation of human monocytes or macrophages^49–51^. Previously, in an independent study using ^1^H nuclear magnetic resonance (NMR)-based untargeted metabolomic approach, our group showed aberrant metabolism in sarcoidosis^52^. In that study, we found increased levels of several intermediates related to fatty acid metabolism and TCA cycle in sera of sarcoidosis subjects^52^. Our current RNA-seq data confirms our previous metabolic data by identifying specific genes involved in mitochondrial β-oxidation, e.g. ACATs.

Our RNA-sequencing results demonstrate that sarcoidosis monocytes exhibit increased gene expression for phagocytosis and lysosomal genes that may result in the increased uptake of extracellular particles and lysosomal degradation. In contrast, we found a significant down regulation of genes involved in proteasome degradation in sarcoidosis monocytes compared to control monocytes. This may indicate that the accumulation of intracellular proteins or phagocytic degradation products may be the cause of the persistent inflammation in sarcoidosis. Further studies need to delineate in detail the functional role of the identified changes in gene expression in sarcoidosis. This dataset will be useful to develop a model organism for sarcoidosis disease or to investigate potential target drugs.

## Methods

### Subjects

The Committee for Investigations Involving Human Subjects at Wayne State University approved the protocol for obtaining blood by phlebotomy from control subjects and patients with sarcoidosis. The IRB number for this study is 055208MP4E. All methods were performed in accordance with the relevant guidelines and regulations. Informed consent was obtained from all subjects enrolled for the study. Sarcoidosis diagnosis was based on the ATS/ERS/WASOG statement^5^. The criteria for enrollment in the diseased group were: (i) a compatible clinical/radiographic picture consistent with sarcoidosis, (ii) histologic demonstration of non-caseating granulomas on the tissue biopsy, and (iii) exclusion of other diseases capable of producing a similar histologic or clinical picture, such as infection by fungus or mycobacteria. Subjects excluded were: (i) smokers, (ii) those receiving immune suppressive medication (defined as corticosteroid alone and/or immune modulatory medications), (iii) those with positive microbial culture in routine laboratory examinations or viral infection; or (iv) those with known hepatitis or HIV infections or any immune suppressive condition. The criteria for enrollment in the control group were**:** (i) absence of any chronic respiratory diseases, (ii) lifetime nonsmoker, (iii) absence of HIV or hepatitis infection. A total of 20 patients with sarcoidosis and 20 controls participated in this study.

### Isolation of PBMCs and purification of monocytes

PBMCs were isolated from heparinized blood using Ficoll-Histopaque (Sigma, St. Louis, MO) density gradient separation method as previously described^19, 20^. CD14 + monocytes were purified from PBMCs by using the MACS monocyte isolation kit (Miltenyl Biotech, San Diego, CA) according to the manufacturer’s instructions. The purity of enriched monocytes was evaluated by flow cytometry using fluorochrome-conjugated CD14 antibody; the purity of monocytes was about 95%.

### mRNA isolation

Purified monocytes were incubated overnight in endotoxin-free RPMI 1640 medium (HyClone) supplemented with L-glutamine (Life Technologies), penicillin/streptomycin (Life Technologies), and FCS (HyClone) at 37 °C and in air containing 5% CO_2_. Cells were washed twice with ice cold PBS and pellets were collected by centrifuging at 2000 rpm. Collected pellets were lysed with Lysis/Binding Buffer (Ambion) and frozen at −80 °C. Poly-adenylated mRNAs were subsequently isolated from thawed lysates using the Dynabeads mRNA Direct Kit (Ambion) following the manufacturer’s instructions.

### RNA-seq library preparation and sequencing

RNA-seq libraries were prepared using the NEBNext ultradirectional library preparation protocol (New England BioLabs, Ipswich, MA). The individual libraries were quantified using the KAPA real-time PCR system, following the manufacturer instructions and using a custom-made series of standards obtained from serial dilutions of the phi-X DNA^53^. Individually barcoded RNA-seq libraries were pooled in equimolar quantities. RNA-seq library quality was assessed using an Agilent Bioanalyzer. A pooled library of 20 samples (10 sarcoidosis monocytes, and 10 healthy monocytes) was sequenced on the Illumina Next-Seq 500 (75 cycles, PE).

### RNA-seq data analysis, differential gene expression, and canonical pathway analysis

RNA-seq data were analyzed using the Illumina Basespace RNA express application (app). In this app, the sequencing reads were aligned to the reference human genome hg19 using STAR aligner and differentially expressed (DE) gene data was obtained with the DEseq2 analysis tool^54, 55^. Gene Trail and iPathwayGuide tools were used to identify the cellular pathways and gene ontology categories that were overrepresented based on the list of DE genes (FDR < 5%). The pathways with FDR < 5% were considered to be significant. The type of FDR adjustment used was Benjamini and Hochberg. Heat maps were generated using the DEseq2 package in R-studio.

### RNA Extraction and Quantitative Reverse Transcriptase/Real Time-PCR (qRT-PCR)

To validate the RNA-seq data, we performed qRT-PCR of selected genes enrolling 10 independent subjects in each study group. RNA was isolated from monocytes of 10 different sarcoidosis patients and 10 healthy controls and qRT-PCR was done as described previously^56^. Relative mRNA expression levels were calculated and normalized to β-actin using delta Ct method. Statistical analysis was done using paired, two-tailed Student’s t-test The following primers were used for PCR reactions: β-actin forward; 5′-CATGTACGTTGCTATCCAGGC 3′and reverse; 5′ CTCCTTAATGTCACGCACGAT-3′, ATP6AP1 forward; 5′-CAAGTCCGAAGATGTCCCATAC-3′ and reverse; 5′- CTTGTACGCCACAGAGAAGTT-3′; CYBB forward; 5′-CTGGACAGGAATCTCACCTTTC-3′ and reverse; 5′-GCCTCCTTCAGGGTTCTTTATT-3′, SerpinA1 forward; 5′-GAAGGTCTGCCAGCTTACAT-3′ and reverse; 5′-GCAGCTTCAGTCCCTTTCT-3′, LAMP2 forward; 5′-CCAACACTACTGGGATGTTCT-3′and reverse, 5′-GCAGACAAGTATCATTGCCATT-3′; RPSA forward; 5′-TAGGTGGCACCAATCTTGAC-3 and reverse; 5′-GCAGCAAACTTCAGCACAG-3, RPL10A forward, 5′-GCACTGTGACGAGGCTAAG-3′ and reverse; 5′-TTGTGTGTGAGCAGGGAAG-3′.

## Electronic supplementary material

Supplementary info
