# Supplementary material for: RNA-sequencing Identifies Novel Pathways in Sarcoidosis Monocytes
Source: Sci Rep. 2017 Jun 2;7:2720. doi: 10.1038/s41598-017-02941-4 (PMC5457404; doi:10.1038/s41598-017-02941-4)
Supplement: Supplementary file 1 — Supplementary info [file 41598_2017_2941_MOESM1_ESM.pdf]

RNA-sequencing Identifies Novel Pathways in Sarcoidosis Monocytes

Jaya Talreja[1], Pershang Farshi[1], Adnan Alazizi [2], Francesca Luca [2,3], Roger Pique-Regi [2,3] and Lobelia Samavati [1,2]

[1]Department of Internal Medicine, Division of Pulmonary, Critical Care and Sleep Medicine, Wayne State University School of Medicine and Detroit Medical Center, Detroit, MI 48201; [2]Center for Molecular Medicine and Genetics, Wayne State University School of Medicine, 540 E. Canfield, Detroit, MI 48201, USA; [3]Department of Obstetrics and Gynecology, Wayne State University School of Medicine, Detroit, MI 48201, USA.

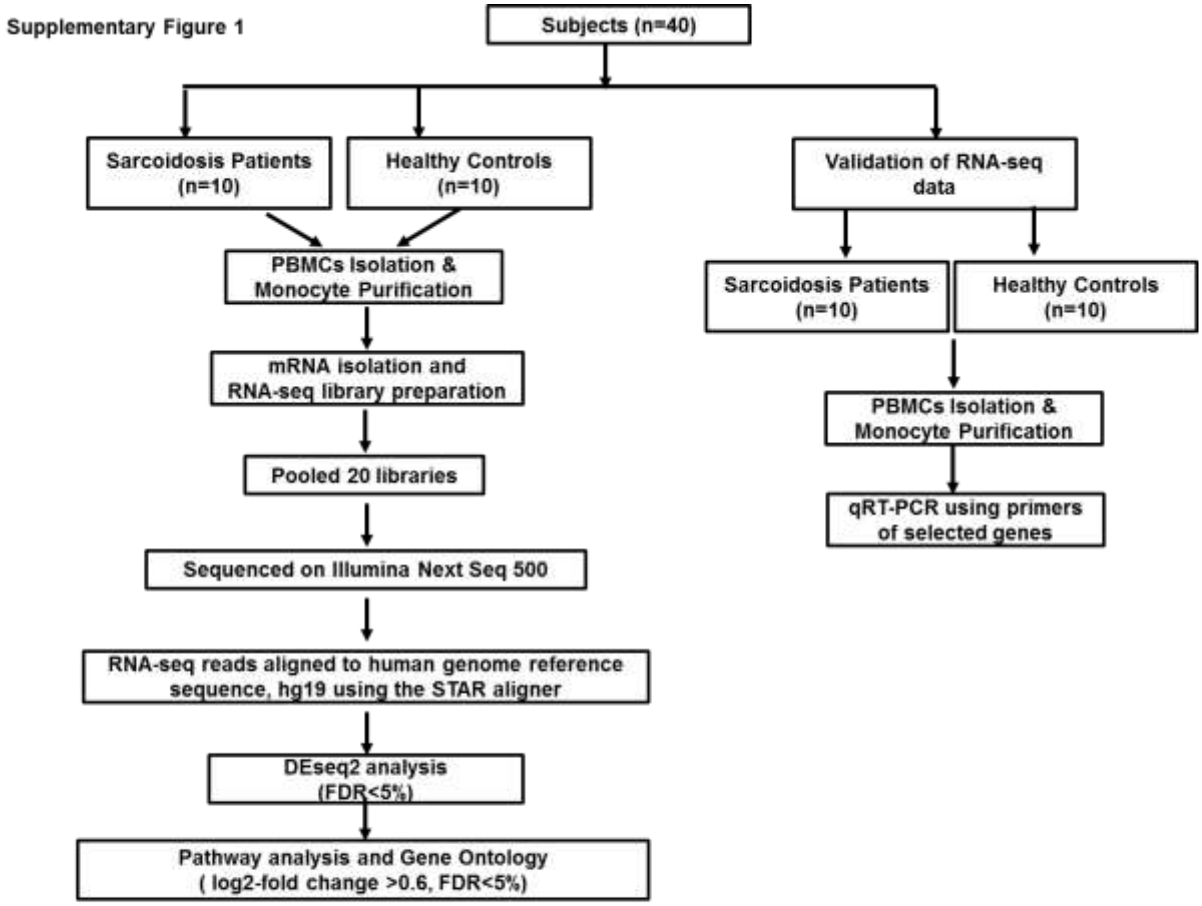

Supplementary Figure Legend

Supplementary Figure S1. Overview of the RNA-seq library preparation and analysis. RNA-seq libraries were prepared from mRNA isolated from monocytes of sarcoidosis patients (n=10) and healthy controls (n=10). Pooled individually barcoded libraries were sequenced on one lane of the Illumina Next-Seq 500 (75 cycles, PE). Reads were mapped against reference human genome hg19 and was analyzed using DEseq2 analysis tool. Functional analysis based on DE genes was done using ipathway guide program tool. RNA-seq data was validated via qRT-PCR using primers for selected genes. Total RNA for qRT-PCR was isolated from monocytes obtained from independent sets of 10 sarcoidosis patients and 10 healthy controls.
